# Sodium Tanshinone IIA Sulfonate Decreases Cigarette Smoke-Induced Inflammation and Oxidative Stress via Blocking the Activation of MAPK/HIF-1α Signaling Pathway

**DOI:** 10.3389/fphar.2018.00263

**Published:** 2018-05-01

**Authors:** Ruijuan Guan, Jian Wang, Ziying Li, Mingjing Ding, Defu Li, Guihua Xu, Tao Wang, Yuqin Chen, Qian Yang, Zhen Long, Zhou Cai, Chenting Zhang, Xue Liang, Lian Dong, Li Zhao, Haiyun Zhang, Dejun Sun, Wenju Lu

**Affiliations:** ^1^State Key Lab of Respiratory Diseases, Guangzhou Institute of Respiratory Disease, Guangzhou Institute of Respiratory Health, The First Affiliated Hospital, Guangzhou Medical University, Guangzhou, China; ^2^Departments of Respiratory and Critical Diseases, Inner Mongolia Autonomous Region People’s Hospital, Hohhot, China; ^3^Department of Clinical Medical Research Center, Inner Mongolia Autonomous Region People’s Hospital, Hohhot, China

**Keywords:** sodium tanshinone IIA sulfonate, COPD, cigarette smoke, hypoxia-inducible factor-1α, inflammation, oxidative stress

## Abstract

Aberrant activation of hypoxia-inducible factor (HIF)-1α is frequently encountered and promotes oxidative stress and inflammation in chronic obstructive pulmonary disease (COPD). The present study investigated whether sodium tanshinone IIA sulfonate (STS), a water-soluble derivative of tanshinone IIA, can mediate its effect through inhibiting HIF-1α–induced oxidative stress and inflammation in cigarette smoke (CS)-induced COPD in mice. Here, we found that STS improved pulmonary function, ameliorated emphysema and decreased the infiltration of inflammatory cells in the lungs of CS-exposed mice. STS reduced CS- and cigarette smoke extract (CSE)-induced upregulation of tumor necrosis factor (TNF)-α and interleukin (IL)-1β in the lungs and macrophages. STS also inhibited CSE-induced reactive oxygen species (ROS) production, as well as the upregulation of heme oxygenase (HO)-1, NOX1 and matrix metalloproteinase (MMP)-9 in macrophages. In addition, STS suppressed HIF-1α expression *in vivo* and *in vitro*, and pretreatment with HIF-1α siRNA reduced CSE-induced elevation of TNF-α, IL-1β, and HO-1 content in the macrophages. Moreover, we found that STS inhibited CSE-induced the phosphorylation of ERK, p38 MAPK and JNK in macrophages, and inhibition of these signaling molecules significantly repressed CSE-induced HIF-1α expression. It indicated that STS inhibits CSE-induced HIF-1α expression likely by blocking MAPK signaling. Furthermore, STS also promoted HIF-1α protein degradation in CSE-stimulated macrophages. Taken together, these results suggest that STS prevents COPD development possibly through the inhibition of HIF-1α signaling, and may be a novel strategy for the treatment of COPD.

## Introduction

Chronic obstructive pulmonary disease (COPD) is a progressive lung malady that affects most of the long-term smokers and is predicted to be the third leading cause of death globally by 2030 (Lundback et al., 2003; Nowrin et al., 2014). It was reported that admission to hospitals owing to COPD accounts most for the direct medical care costs in many developed countries (Foster et al., 2006). However, there is no effective treatment for COPD, and its global prevalence is gradually rising (Yu et al., 2012). Hence, there is an enormous need to explore the pathogenesis of COPD and to prevent and treat it.

Chronic obstructive pulmonary disease (COPD) is characterized by emphysema and pulmonary function decline that is usually associated with an aberrant inflammatory response within the airways and lungs to noxious gasses and/or particles (Chung and Adcock, 2008; Kang and Shadel, 2016). Inflammation, starting prior to the onset of clinical symptoms, is seen throughout the bronchial tree and parenchyma of lungs from animals and patients with COPD (Kang and Shadel, 2016; Zhou et al., 2016). It has been recognized as the primary determinant of multimorbidities in COPD patients (Niewoehner et al., 1974). Additionally, these activated inflammatory cells including macrophages can release enormous amounts of reactive oxygen species (ROS) and thus induces oxidative stress, resulting in activation of metalloproteases and lung cell death (Han et al., 2011). Therefore, effective control of inflammation and oxidative stress in animals and patients with COPD is becoming very crucial.

Hypoxia-inducible factor (HIF)-1, a heterodimer complex belongs to the basic helix-loop-helix transcription factors, is composed of HIF-1α and HIF-1β and activates the transcription of target genes under hypoxia conditions (Wang et al., 1995; Terzuoli et al., 2010). Previous studies have shown that HIF-1α protein synthesis was regulated through the PI3K/AKT and MAPK pathways, and its expression is regarded as the suppression factor for HIF-1 DNA binding and transcriptional activities in many cultured cells (Frede et al., 2006; Olson and van der Vliet, 2011; Yu et al., 2012). Another studies also demonstrated that HIF-1α contributes to the development of myeloid cell–mediated inflammation, facilitates the phagocytic function of macrophages and exacerbates the acetaminophen-induced oxidative stress and hepatotoxicity (Cramer et al., 2003; Peyssonnaux et al., 2005; Li et al., 2017). Recently, HIF-1α was demonstrated to be increased in the lungs of COPD patients and activation of HIF-1α signaling pathway by cigarette smoke (CS), a key pathological driver of COPD, accelerates the development of COPD (Ding et al., 2015; Baz-Davila et al., 2016). Thus, pharmacological intervention that inhibits the HIF-1α–mediated inflammation and oxidative stress in macrophages might be beneficial in the prevention and treatment of tissue damage associated with prolonged inflammation.

**FIGURE 1:**
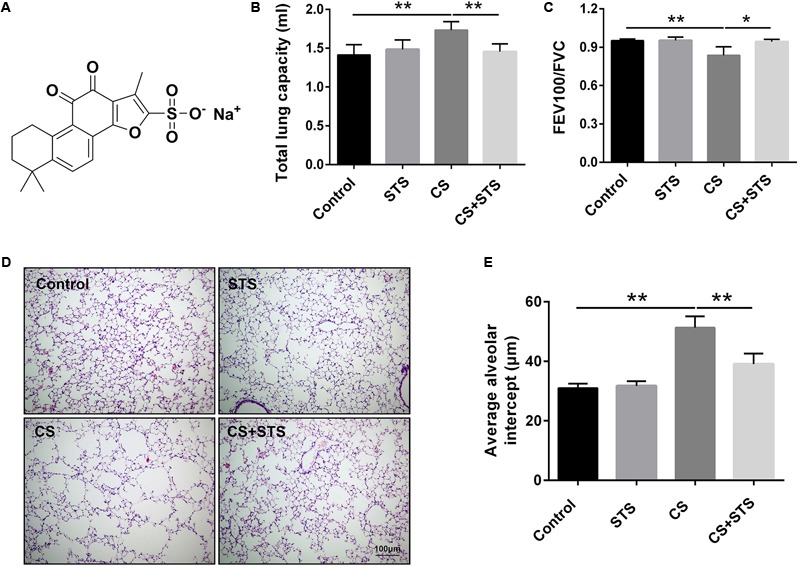
STS ameliorated cigarette smoke (CS)-induced COPD in mice. **(A)** Chemical structure of STS. **(B,C)** 3 months after CS inhalation, lung function parameters including total lung capacity and FEV100/FVC among different groups were calculated. **(D)** Lung pathology was determined by H&E staining. Scale bars = 100 μm. **(E)** Average alveolar intercept, representing the degree of emphysema was quantified by Image Pro Plus 6.0 software. Data are expressed as mean ± SEM, *n* = 6, *^∗^P* < 0.05; *^∗∗^P* < 0.01.

Danshen, an important herbal medicine derived from the dried root and rhizome of *Salvia miltiorrhiza* Bunge, has been clinically used for the treatment of miscellaneous inflammatory conditions associated with cardiac and lung diseases in Southeastern Asian countries for thousands of years (Yang et al., 2008; Zhang et al., 2014). Sodium tanshinone IIA sulfonate (namely STS, **Figure 1A**) is a water-soluble derivative of tanshinone IIA which is one of the key components of danshen (Jiang et al., 2016). STS or tanshinone IIA was demonstrated to have multiple pharmacological properties such as anti-oxidative, anti-tumor and anti-inflammatory activities (Huang et al., 2015; Li et al., 2016; Cheng et al., 2017). Previous studies suggested that tanshinone IIA reduced HIF-1α–mediated inflammatory response in LPS-induced lung injury (Xu et al., 2011). Whether STS can reduce CS-induced inflammation and oxidative stress in COPD mice and whether the protective effect of STS is correlated with HIF-1α expression are still not clear.

In this study, we demonstrated that STS improved the pulmonary function and emphysema, and alleviated CS-induced inflammation in COPD mice. STS inhibited cigarette smoke extract (CSE)-induced inflammation and oxidative stress in macrophages. Moreover, we demonstrated that the protective effects of STS are associated with the inhibition of CS-induced HIF-1α expression *in vitro* and *in vivo*. Hence, our results provide a rationale for the treatment of COPD using STS.

## Materials and Methods

### Animals and Experiment Design

Male C57BL/6J mice, weighting 18–20 g, were purchased from Nanjing BioMedical Research Institute of Nanjing University (Nanjing, China), and allowed free access to food and water in a restricted pathogen-free room with temperature-controlled temperature (25°C) under 12 h light/12 h dark cycle. All animal experiments were performed according to the Criteria of the Medical Laboratory Animal Administrative Committee of Guangdong and the Guide for Care and Use of Laboratory Animals of Guangzhou Medical University, and the protocols were approved by the Ethics Committee for Experimental Research, The First Affiliated Hospital, Guangzhou Medical University. The mice were randomly divided into four groups: Control, STS, CS, and STS + CS groups. To establish the COPD mouse model, mice in the CS and CS + STS groups were treated with Lipopolysaccharide (LPS, 7.5 μg in 50 μl saline, Sigma-Aldrich, St Louis, MO, United States) via tracheal instillation on the 1st and 14th days, and then exposed to CS that were produced by 9 commercially filtered cigarettes (Guangdong Tobacco Industry Co., Ltd., Guangdong, China) for 4 h per day and 6 days per week in a whole body exposure chamber for 3 months except for the days for LPS treatment. For the drug therapy, mice in the STS and CS + STS groups were given STS (5 mg/kg, twice per day) by airway inhalation with a PARI Nebuliser in a whole-body exposure chamber before being exposed to CS. The STS was purchased from Chengdu Herbpurify Co., Ltd (Chengdu, China), and its purity was greater than 98% by HPLC analysis. Meanwhile, mice in the Control and CS groups were given an equal amount of saline instead of STS. Three months after CS inhalation, the mice were sacrificed and used to study the effect of STS on COPD.

### Pulmonary Function Tests

Pulmonary function tests were performed as described previously by Ma et al. (2017). The total lung capacity (TLC) and FEV100/FVC were obtained according to the Buxco resistance/compliance application manual.

### Bronchoalveolar Lavage

Mice were sacrificed with 1% pentobarbital sodium (50 mg/kg, i.p.). Then the lungs underwent lavage with 0.6 ml saline for three times. Bronchoalveolar lavage fluid (BALF) was collected and centrifuged (800 × *g*, 5 min, 4°C). Then the sediment cells were resuspended with 1ml saline for cell classification and counting.

### Lung Pathological Analysis

Left lungs were perfused with 10% buffered formalin and then immediately immersed and fixed in this fixative solution for 24 h. The lung tissues were embedded in paraffin, cut into 3-μm-thick sections and then stained with hematoxylin and eosin (H&E). The average alveolar intercept of the lungs was measured by Image J.

### Immunohistochemistry

Immunohistochemistry assay was performed according to the method described previously (Guan et al., 2016a). The primary antibodies used in our study were as follows: mouse anti- interleukin (IL)-1β antibody (1:800, Servicebio, Wuhan, China).

### ELISA

Lung tissues and the supernatant from macrophage RAW 264.7 cells were prepared for ELISA. The lung issues were homogenated with PBS and then centrifuged (12000 rpm, 15 min, 4°C) to obtain supernatants. The concentration of tumor necrosis factor (TNF)-α and IL-1β was measured using the ELISA kit following the manufacturer’s protocol (eBioscience Affymetrix, Santa Clara, CA, United States).

### Preparation of Cigarette Smoke Extract

CSE was prepared by bubbling smoke from two cigarettes (1.3 mg of nicotine, 13 mg of tar, and 15 mg of carbon monoxide per cigarette) into 10 ml Dulbecco’s modified Eagle’s medium (DMEM) according to the method described by Li et al. (2012).

### Cell Culture and Treatment

Mouse macrophage RAW 264.7 cells were obtained from Cell Bank of the Chinese Academy of Sciences (Shanghai, China), and cultured in DMEM containing 10% fetal bovine serum, 100 U/mL penicillin and 100 mg/L streptomycin in a humidified incubator at 37°C with 5% CO_2_ atmosphere. Macrophages were plated in a six-well plate (Corning, Corning, NY, United States) and treated with STS or different types of inhibitors in the presence and absence of 2% CSE. All the culture reagents used in our study were purchased from Gibco (Carlsbad, CA, United States). Each cell experiment was repeated three times.

### Cell Viability Assay

Macrophages were seeded in a 96-well plate and stimulated with different concentrations of STS as indicated. Cell viability was examined using a CCK-8 kit (Dojindo, Japan) according to the manufacturer’s instructions.

### Measurement of Intracellular ROS

Intracellular ROS production was detected by staining RAW 264.7 cells with an oxidation-sensitive fluorescent probe 2,7-dichlorodihydrofluorescein diacetate (DCFH-DA) according to the manufacturer’s protocol. RAW 264.7 cells were treated with CSE and/or STS for 24 h at 37°C. Subsequently, the medium was removed, and the cells were incubated with 1ml of DCFH-DA (Beyotime Institute of Biotechnology, Haimen, China) solution 37°C for 20 min. The dye solution was then removed, and the cells were trypsinized and centrifuged at 500 × *g* for 5 min at 4°C. Finally, the cells were washed twice with PBS, and kept in the dark on ice. The fluorescence intensity was measured with a microplate reader at 488 nm excitation and 525 nm emission wavelengths.

### HIF-1a Knock-Down

The mouse HIF-1α siRNA was purchased from GenePharma (Suzhou, Jiangsu, China). RAW 264.7 cells were transfected with HIF-1α siRNA by using GenMute^TM^ siRNA Transfection Reagent (SignaGen, United States) in accordance to the manufacturer’s instructions.

### Quantitative Real-Time PCR (RT-PCR)

Total RNA was extracted from lung tissues using TRIzol Reagent (Life Technologies, Carlsbad, CA, United States) and reverse-transcribed into first-strand cDNA using the PrimeScript RT reagent Kit with gDNA Eraser (TAKARA, Japan). The mRNA levels of TNF-α, IL-1β, and HIF-1α were analyzed with an iCyler iQ RT-PCR Detection System (Bio-Rad Laboratories Inc., United States). Relative levels of mRNA expression were normalized to Actin expression for each gene. The target gene and their primer sequences are shown as follows: mouse TNF-α (Forward: 5′-CCCTCCTGGCCAACGGCATG-3′, Reverse: 5′-TCGGGGCAGCCTTGTCCCTT-3′); mouse IL-1β (Forward: 5′-GCCTCGTGCTGTCGGACCCATAT-3′, Reverse: 5′-TCCTTTGAGGCCCAAGGCCACA-3′), mouse HIF-1α (Forward: 5′-GATGACGGCGACATGGTTTAC-3′, Reverse: 5′-CTCACTGGGCCATTTCTGTGT-3′); mouse HO-1 (Forward: 5′-GATAGAGCGCAACAAGCAGAA-3′, Reverse: 5′-CAGTGAGGCCCATACCAGAAG-3′); mouse MMP-9 (Forward: 5′-CTACATAGACGGCATCCAG-3′, Reverse: 5′-CTGTCGGCTGTGGTTCAGT-3′); mouse β-actin (Forward: 5′-CGTGCGTGACATCAAAGAGAAG-3′, Reverse: 5′-CCAAGAAGGAAGGCTGGAAAA-3′).

### Western Blot

Western blot analysis was performed as described previously (Guan et al., 2016b). The primary antibodies used in our study were as follows: mouse anti-HIF-1α antibody (1:1000, Servicebio, Wuhan, China); Rabbit anti-IL-1β antibody (1:1000, Abcam Biotechnology, Cambridge, MA, United States); Rabbit anti-p-ERK, anti-ERK, anti-p-JNK, anti-JNK, anti-p-P38 and anti-P38 antibodies (1:2000, Cell Signaling Technology, Danvers, CA, United States); Rabbit anti-heme oxygenase (HO)-1 and anti-Nox1 (1:3000, Abcam Biotechnology, Cambridge, MA, United States); Rabbit anti-matrix metalloproteinase (MMP)-9 (1:1000, Proteintech, Chicago, IL, United States); Mouse anti-actin polyclonal antibody (1:3000, Abcam Biotechnology, Cambridge, MA, United States).

### Data Analysis

Statistical analyses were determined using SigmaPlot Software (Systat, United States). Student’s *t*-test was applied to comparisons between two groups and one-way ANOVA was used for comparisons between more than two groups. Data were presented as mean ± SEM. A *p*-value of <0.05 was regarded significant.

## Results

### STS Improved Pulmonary Function and Emphysema in CS-Exposed Mice

To investigate the effects of STS on CS-induced COPD in mice, lung function measurement was performed. The results showed that the pulmonary function of STS-treated mice was significantly improved, as demonstrated by decreased TLC and increased FEV100/FVC value, when compared with CS-exposed mice (**Figures 1B,C**). Further, H&E staining of the lungs was also performed. We found that 3 months after CS inhalation, the average alveolar intercept in the lungs of mice exposed to CS are much larger than those exposed to normal room air, implying that CS inhalation successfully caused emphysema in mice. However, STS intervention significantly reduced the increase in the average alveolar intercept of the lungs exposed to CS for 3 months, indicating that STS ameliorated CS-induced emphysema in mice (**Figures 1D,E**). STS in non-CS-exposed mice generated no changes in lung function or pulmonary pathology.

### STS Inhibited CS-Induced Inflammation in the Lungs

**FIGURE 2:**
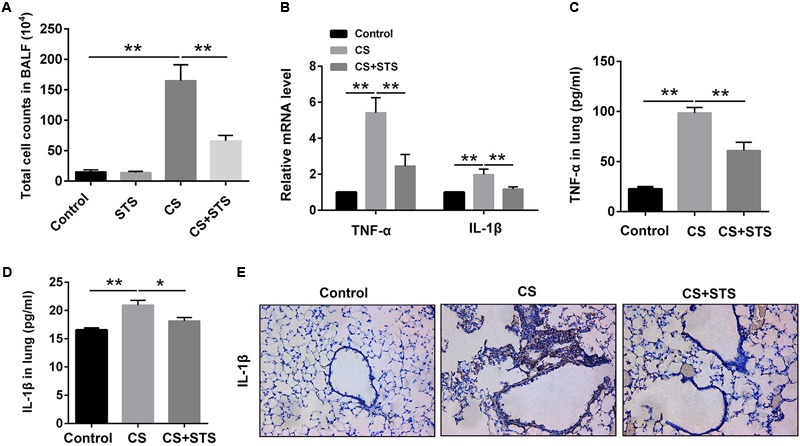
STS reduced the numbers of inflammatory cells and inflammatory factors in the CS-induced COPD in mice. Three months after CS inhalation, lung tissues and BALF was collected. **(A)** The total cells in BALF were calculated. **(B)** The mRNA levels of TNF-α and IL-1β in the lung tissues were analyzed by Quantitative real-time PCR (RT-PCR). **(C,D)** The relative protein levels of TNF-α and IL-1β in the lung tissues were analyzed by ELISA. **(E)** Immunohistochemical staining of IL-1β-positive cells in the lungs. Data are presented as mean ± SEM, *n* = 6, ^∗^*P* < 0.05; ^∗∗^*P* < 0.01.

Accumulating evidences suggest that inflammatory response plays a key role in the pathological mechanism of COPD (Ma et al., 2017). To evaluate the effects of STS on the CS-induced pulmonary inflammation responses, the total cell counts in BALF were measured. The data showed that CS inhalation increased the number of inflammatory cells, while STS treatment markedly reduced inflammatory cell recruitment in the lungs on day 90 (**Figure 2A**). Inflammatory cytokines such as TNF-α and IL-1β, secreted mainly by inflammatory cells, have been well-documented to play crucial roles in the pathogenesis of COPD. We, therefore, examined the levels of TNF-α and IL-1β in the lungs in our study. The results showed that TNF-α and IL-1β were marginally expressed in the lungs from the two control groups. Three months after CS inhalation, the mRNA and protein levels of TNF-α and IL-1β were all increased, as compared with the control group. STS treatment reduced the CS-induced expression of TNF-α and IL-1β in the lungs (**Figures 2B–D**). Similarly, our immunohistochemistry studies showed that the IL-1β-positive cells were also reduced by STS treatment (**Figure 2E**).

### STS Inhibited CSE-Induced Inflammation Cytokines in RAW 264.7 Macrophages

**FIGURE 3:**
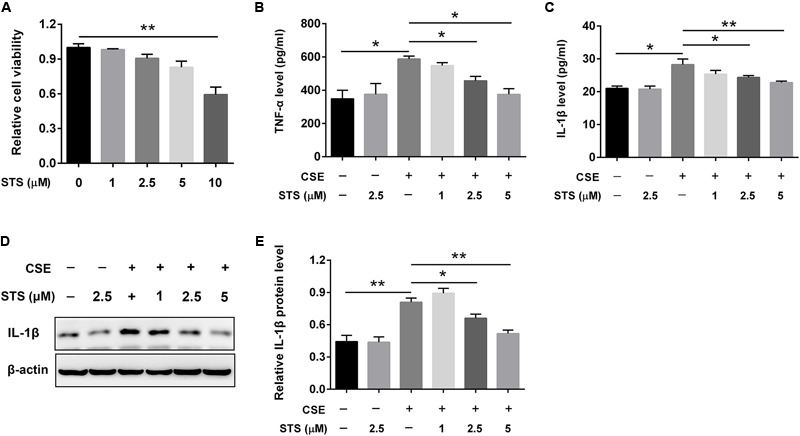
STS inhibited CSE-induced production of inflammation cytokines in RAW 264.7 macrophages. RAW 264.7 macrophages were cultured with and without CSE and/or 1, 2.5, or 5 μM STS for 24 h. **(A)** The viabilities of macrophages treated as indicated were measured by CCK-8 assay. **(B,C)** The supernatant was collected to detect the content of TNF-α and IL-1β by ELISA. **(D,E)** The protein level of IL-1β in the macrophages was analyzed by Western blot. Data are presented as mean ± SEM, *n* = 3, ^∗^*P* < 0.05; ^∗∗^*P* < 0.01.

Since macrophages are potentially very critical effector cells in COPD (Barnes, 2004), we also determined the levels of these two cytokines in macrophage cell line (RAW 264.7 cell). Initially, we evaluated the cytotoxicity of STS in macrophages. The CCK-8 assay showed that STS at the concentrations of 1, 2.5, and 5 μM induced no significant cell cytotoxicity (**Figure 3A**). We then investigated whether STS inhibited CSE-induced inflammatory reaction in macrophages under the above concentrations. The results showed that the protein level of TNF-α and IL-1β was significantly elevated in the supernatant from macrophages and this elevation was inhibited by STS treatment, as examined by ELISA (**Figures 3B,C**). Consistently, Western blot analysis showed that the IL-1β protein level was also significantly increased in CSE-treated macrophages. In contrast, STS-treated macrophages also exhibited reduced IL-1β protein level in a dose-dependent manner when compared with CSE-stimulated macrophages (**Figures 3D,E**).

### STS Attenuated CSE-Induced Oxidative Stress and MMP-9 Upregulation in Macrophages

**FIGURE 4:**
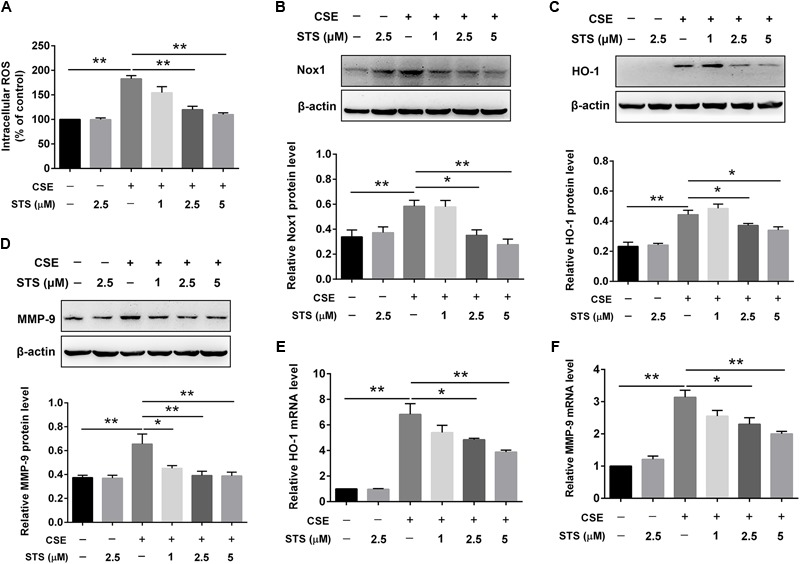
STS inhibited CSE-induced oxidative stress and MMP-9 upregulation in macrophages. RAW 264.7 macrophages were treated with CSE and different concentrations of STS (1, 2.5, and 5 μM) for 24 h. **(A)** The intracellular ROS was determined using a fluorescence microplate according to the ROS assay kit. The relative protein levels of NOX1 **(B)**, HO-1 **(C)**, and MMP-9 **(D)** were determined by Western blot. The mRNA levels of HO-1 **(E)** and MMP-9 **(F)** in the lung tissues were analyzed by RT-PCR. Data are presented as mean ± SEM, *n* = 3, ^∗^*P* < 0.05; ^∗∗^*P* < 0.01.

The activated inflammatory cells including macrophages can induce oxidative stress, resulting in activation of metalloproteases and lung cell death (Han et al., 2011). We therefore investigated the effects of STS on oxidative stress and MMP-9 expression in CSE-stimulated macrophages. Oxidation reactions by ROS are regarded as a trigger of the oxidative stress (Li et al., 2011). Thus, we firstly examined the effects of STS on intracellular ROS production in RAW 264.7 cells. As shown in **Figure 4A**, CSE significantly increased ROS level in macrophages, however, this was attenuated by STS treatment. Additionally, Western blot analysis demonstrated that the protein levels of NOX1 and HO-1 were significantly increased in the CSE-stimulated macrophages, further suggesting the activation of oxidative stress in macrophages. While STS-treated macrophages exerted reduced protein levels of NOX1 and HO-1 when compared with CSE-stimulated macrophages, further indicating the inhibitory effect of STS on oxidative stress (**Figures 4B,C**). Consistent with its suppression of HO-1 protein expression, the CSE-induced increase of HO-1 mRNA level was also significantly inhibited by STS (**Figure 4E**). Moreover, the upregulation of MMP-9 mRNA and protein levels in macrophages caused by CSE was also inhibited by STS treatment (**Figures 4D,F**).

### STS Suppressed CS-Induced HIF-1α Expression in the Lungs and Macrophages

**FIGURE 5:**
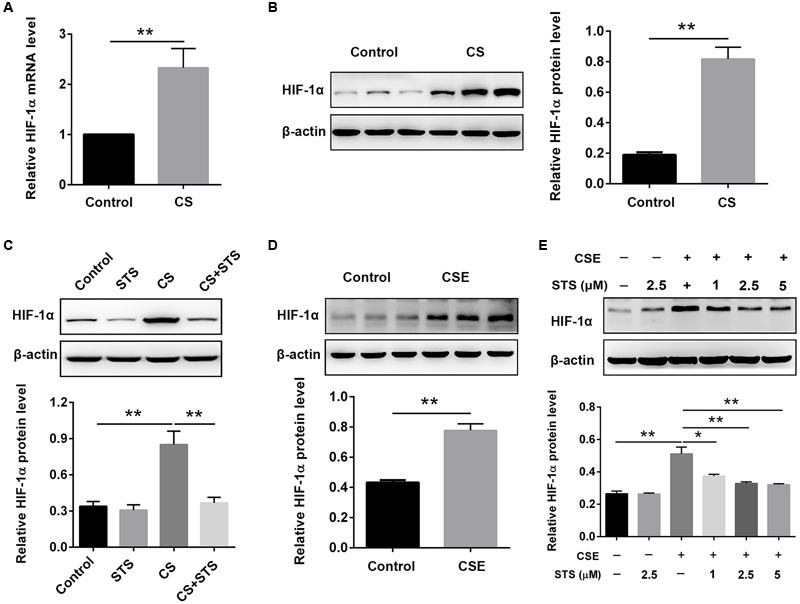
STS suppressed CS-induced HIF-1α expression in the lungs and in macrophages. **(A–C)** 3 months after CS inhalation, lung tissues were collected and prepared for RT-PCR and Western blot analysis using antibodies against HIF-1α and β-actin. Data are presented as mean ± SEM, *n* = 6, ^∗∗^*P* < 0.01. **(D,E)** Macrophages were treated with CSE and different concentrations of STS (1, 2.5, and 5 μM) for 24 h. Cells were then collected and prepared for Western blot analysis using antibodies against HIF-1α and β-actin. Data are presented as mean ± SEM, *n* = 3, ^∗^*P* < 0.05; ^∗∗^*P* < 0.01.

Real-time PCR (RT-PCR) and Western blot results showed that the mRNA and protein levels of HIF-1α were dramatically increased, suggesting the activation of HIF-1α signaling in COPD (**Figures 5A,B,D**). Whereas treatment with STS significantly decreased the CS-induced up-regulation of HIF-1α protein (**Figure 5C**). Coincidentally, the enhancement of HIF-1α expression induced by CSE in RAW 264.7 macrophages was also significantly suppressed by STS treatment in a dose-dependent manner (**Figure 5E**).

### STS Repressed CSE-Induced Elevation of Inflammation Cytokines and HO-1 Expression via Inhibition of HIF-1α in Macrophages

**FIGURE 6:**
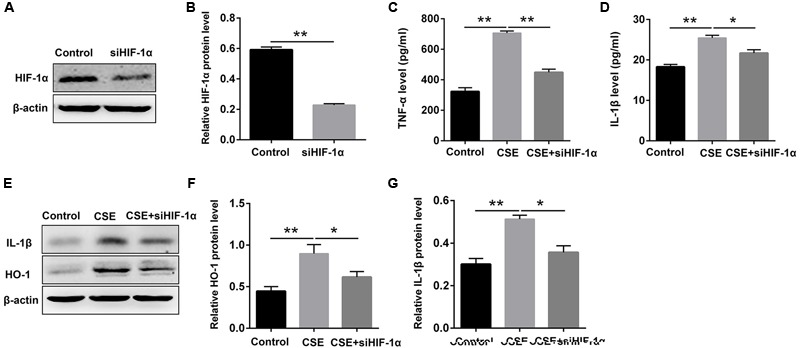
HIF-1α siRNA repressed CSE-induced elevation of inflammation cytokines and HO-1 expression in macrophages. Macrophages were transfected with HIF-1α siRNA by using Lipofectamine 2000 according to the manufacturer’s instructions. **(A,B)** 6 h after transfection, Western blot analysis was performed to evaluate the changes of HIF-1α expression. **(C,D)** Transfected macrophages were deprived of FCS for 6 h and incubated with CSE for 24 h. The supernatant was collected to examine the content of TNF-α and IL-1β by ELISA. **(E–G)** Transfected macrophages were deprived of FCS for 6 h and incubated with CSE for 24 h. The cell samples were collected to measure the content of IL-1β and HO-1 by Western blot. Data are presented as mean ± SEM, *n* = 3, ^∗^*P* < 0.05; ^∗∗^*P* < 0.01.

It has been demonstrated that HIF-1α plays a critical role in regulating CS-mediated inflammation response (Jiang et al., 2010; Eurlings et al., 2014). We then determined whether the inhibition of inflammation by STS was associated with the suppression of HIF-1α content. The results showed that HIF-1α siRNA substantially reduced HIF-1α expression in macrophages, approximately 61.4% (**Figures 6A,B**), suggesting an effective knockdown of HIF-1α by siRNA. Moreover, HIF-1α siRNA caused a reduction in TNF-α, IL-1β and HO-1 content in the macrophages treated with CSE for 24 h, suggesting that the suppression of CSE-induced HIF-1α expression might contribute to the inhibition of inflammation and oxidative stress by STS (**Figures 6C–G**).

### STS Inhibited CSE-Induced HIF-1α Expression via Inactivation of MAPK Signaling in Macrophages

**FIGURE 7:**
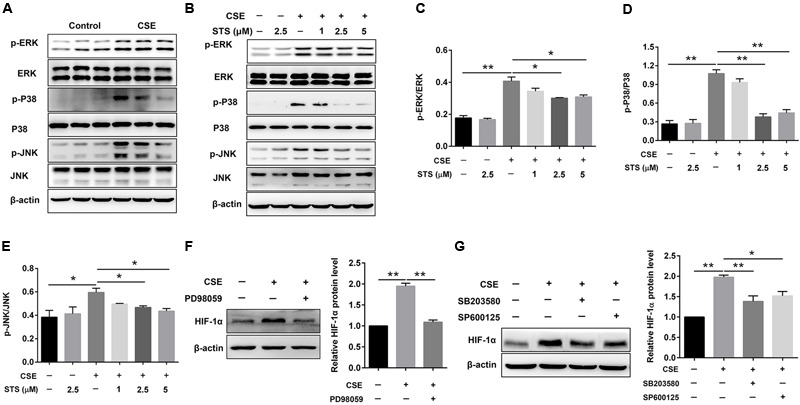
STS inhibited CSE-induced MAPK activation in macrophages. RAW 264.7 macrophages were treated as indicated for 24 h. Cell lysates were prepared and immunoblotted with antibodies for p-ERK, p-P38 and p-JNK. **(A,B)** Western blot was used to analyze the phosphorylation levels of p-ERK, p38 MAPK and JNK. Results are representative of different experiments. **(C–E)** Scanning densitometry of western blot on different samples was analyzed quantitatively. Expression of p-ERK, p-P38 and p-JNK was normalized to ERK, P38 MAPK and JNK level, respectively. **(F,G)** The relative protein level of HIF-1α was determined by Western blot. Data are presented as mean ± SEM, *n* = 3, ^∗^*P* < 0.05; ^∗∗^*P* < 0.01.

Western blot analysis showed that CSE stimulation significantly increased the phosphorylation of ERK, p38 MAPK and JNK in macrophages (**Figure 7A**), suggesting the activation of MAPK signaling. The phosphorylation of ERK, p38 MAPK and JNK was markedly down-regulated by STS, indicating the suppression of MAPK signaling (**Figures 7B–E**). In addition, treatment with PD98059 (ERK inhibitor), SB203580 (p38 MAPK inhibitor) or SP600125 (JNK inhibitor) also significantly inhibited CSE-induced HIF-1α expression (**Figures 7F,G**). Taken together, these data indicated that the suppression of ERK, p38 MAPK and JNK activation by STS has been involved in the down-regulation of CSE-induced HIF-1α expression.

### STS Promoted CSE-Induced HIF-1α Protein Degradation in Macrophages

**FIGURE 8:**
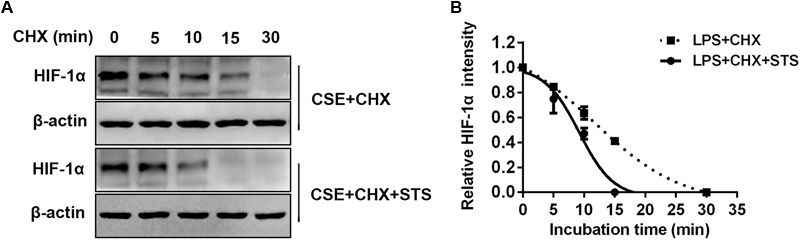
STS promoted CSE-induced HIF-1α protein degradation in macrophages. RAW 264.7 macrophages were treated with CSE for 24 h with or without 5 μM STS, followed by incubation with cycloheximide (CHX) from 0 to 30 min. **(A)** Cell lysates were then collected and prepared for Western blot analysis using antibodies against HIF-1α or β-actin. **(B)** The relative intensity of HIF-1α protein content was quantified. Data are presented as mean ± SEM, *n* = 3.

In addition to the suppression of HIF-1α protein synthesis, whether STS affects HIF-1α protein degradation remains unknown. We then investigated the effect of STS on the stability of HIF-1α protein in RAW 264.7 cells. Cells were treated with or without STS (5 μM) in the presence of CSE stimulation for 24 h, followed by incubation with cycloheximide (CHX) for different times (0–30 min) to block ongoing protein synthesis. As shown in **Figure 8**, in the presence of CHX, the half-life of HIF-1α protein in the macrophages treated with STS was significantly shorter than that in macrophages treated without STS. Our data showed that STS also promoted HIF-1α protein degradation.

## Discussion

In the present study, we investigated the possible role of STS in the development of COPD and determined whether STS exerted its anti-oxidative and anti-inflammatory effects via the blockade of HIF-1α signaling pathway. The results showed that STS effectively improved pulmonary function and emphysema in the COPD mice. STS decreased CS-induced inflammatory response and oxidative stress both in the *in vivo* and *in vitro* models of COPD and identified that the protective effect of STS was related to the inhibition of CS-induced HIF-1α accumulation. In addition, STS repressed CSE-induced ERK, p38 MAPK and JNK activation in macrophages, indicating that ERK, p38 MAPK and JNK inactivation mediated the STS-induced effects on CSE-induced oxidative stress and inflammation. Furthermore, we demonstrated that STS affected CSE-induced HIF-1α protein synthesis via the suppression of ERK, p38 MAPK and JNK signaling pathways. Simultaneously, we demonstrated that STS also promoted HIF-1α protein degradation.

Chronic obstructive pulmonary disease (COPD) is a chronic, progressive and ultimately lethal pulmonary disease with unknown etiology (Pauwels et al., 2001). It encompasses a range of clinical syndromes, most remarkably emphysema and chronic inflammation (Hogg and Timens, 2009). COPD arises from genetics or environmental exposure (Chung and Adcock, 2008). However, smoking is regarded as the primary contributing factor of COPD, especially in those developing countries where smoking prevalence is increasing (Hogg et al., 2004). COPD caused by CS is a major and leading cause of morbidity and mortality worldwide (Mannino and Buist, 2007). Thus, the CS-induced mouse model is the most useful model of COPD. Previous studies have demonstrated that pretreatment with LPS can accelerate the inflammatory response and simulate the pathologic process of COPD (Hardaker et al., 2010). To create a COPD-like mouse model in a shorter exposure period, exposing mice to a combination of CS and LPS for 3 months is a well-established method (Zhou et al., 2012). In the present study, we also adopted this model to inquire into the effect and latent mechanisms of STS in COPD. Our results showed that CS induced obvious COPD, as evidenced by the decreased lung function and increased pulmonary inflammation and emphysema. These results revealed that we had successfully built a COPD mouse model. Interestingly, we observed a marked improvement of emphysema and pulmonary function in these CS-exposed mice after STS treatment. Further, STS was well tolerated by the mice as indicated by no discernible signs of toxicity. Thus, our study firstly confirmed that this traditional medicine reduced disease progression and might be used to treat COPD in the future.

Chronic obstructive pulmonary disease (COPD) is characterized by airflow limitation and emphysema that is usually associated with a turgescent chronic inflammatory response in the airways and the lung (Chung and Adcock, 2008). Inflammation with infiltrating various inflammatory cells such as macrophages, neutrophils and lymphocytes, is a pivotal contributing factor in the COPD development and is believed to be closely related to emphysema and pathologic alterations, which further worsen with this disease progression (Niikura et al., 2015; Kang and Shadel, 2016). Hence, we firstly investigated the effect of STS on CS-induced inflammatory response. The results showed that CS inhalation induced an increase in the total number of inflammatory cells in BALF and this upregulation was inhibited by STS intervention, implying that STS reduced the infiltration of leukocytes to the lungs. The decline of inflammation with STS treatment we observed is in conformity with previous studies demonstrating that STS has an anti-inflammatory role in LPS-induced lung injury (Xu et al., 2011; Zhang et al., 2014). The momentous biological mediators of COPD are pro-inflammatory cytokines including TNF-α and IL-1β, which are found to be memorably increased in COPD patients and released by massive inflammatory cells in response to CS treatment (Rahman and Adcock, 2006). Then, the effect of STS on the production of these inflammatory cytokines was investigated. We found that treatment with STS significantly decreased the enhanced production of TNF-α and IL-1β in mice induced by CS. We also found that STS markedly reduced the production of these cytokines in RAW 264.7 macrophages, which are very important effector cells in COPD, in a dose-dependent manner, resembling several previous studies in activated RAW 264.7 cells (Jang et al., 2003; Barnes, 2004; Fan et al., 2009). Taken together, these findings clearly suggest that STS might exert its anti-inflammatory effect via the inhibition of TNF-α and IL-1β production in macrophages.

In addition to producing pro-inflammatory cytokines, the activated inflammatory cells including macrophages also induce oxidative stress, which can directly undermine cellular components such as proteins, lipids and DNA, resulting in activation of metalloproteases and lung cell death (Barnes et al., 2003; Szilasi et al., 2006). It has been reported that oxidative stress occurs when ROS are produced in excess of the antioxidant capacity and the increased burden of oxidative stress correlates conversely with lung function and, for ages, oxidative stress due to the imbalance between oxidants and antioxidants has been identified as a basis for COPD (Han et al., 2011; Kang and Shadel, 2016). Among ROS-producer enzymes, the NADPH oxidase family, including NOX associated regulatory subunits, has been demonstrated to lie behind lung damage found in COPD (Trocme et al., 2015). HO-1 can degrade heme under exogenous oxidative insults including CS and is therefore considered as a stress-induced enzyme (Cui et al., 2017). Consistent with these findings, our results showed that both the intracellular ROS and the protein expression of NOX1 and HO-1 were significantly increased after CSE stimulation, and treatment with STS suppressed the intracellular ROS production and the increased expression of NOX1 and HO-1, implying the inhibitory role of STS in oxidative stress. Moreover, macrophages also produce excessive of proteases including MMPs, which are able to damage the elastin and other components of the alveolar wall followed by emphysema and decline in pulmonary function (Russell et al., 2002). Impressively, our data also showed that the enhancement of MMP-9 in macrophages response to CSE stimuli was significantly inhibited by STS treatment. This further explains why STS can attenuate CS-induced emphysema.

The mechanism by which STS suppresses pulmonary inflammation and oxidative stress remains obscure. Cumulative evidences implicate the presence and activation of HIF-1α transcription factor in a variety of inflammatory diseases including CS-induced COPD, and recent studies have signified its critical importance in regulating the phagocyte function and inflammatory mediator production (Olson and van der Vliet, 2011; Ding et al., 2015). HIF-1α activation inhibits neutrophil apoptosis and subsequent inhibits phagocytosis by macrophages. While HIF-1α deficient in myeloid cells caused nearly complete suppression of inflammatory response, as indicated by attenuated inflammatory cell infiltration and edema (Cramer et al., 2003; Olson and van der Vliet, 2011). Therefore, we hypothesized that STS can exert its anti-inflammatory effect via inactivation of HIF-1α in our COPD mouse model. As expected, the results showed that the CS-induced increase of HIF-1α expression in the lungs was remarkably suppressed by STS. This finding was also validated in CSE-primed RAW 264.7 macrophages, which is similar to a previous study that tanshinone IIA inhibited HIF-1α expression in LPS-induced lung injury (Xu et al., 2011). Hence, we confirmed that STS might play a therapeutic role in COPD by blocking HIF-1α activation. For further verification, we also used HIF-1α siRNA to observe the effect of HIF-1α on the production of cytokines in macrophages, and found that the HIF-1α siRNA significantly inhibited the expression of TNF-α and IL-1β induced by CSE. Our results are partially in accordance with a previous study by Xu et al. (2011) who demonstrated that HIF-1α contributes to LPS-induced production of inflammatory factor such as TNF-α and IL-1β in NR8383 cells (Xu et al., 2011). Coincidentally, HIF-1α siRNA also significantly repressed the expression of HO-1, conforming that HIF-1α was involved in CSE-induced oxidative stress. Hence, we concluded that STS might play a therapeutic role in COPD by blocking HIF-1α activation.

We then investigated the mechanisms whereby STS interfered with HIF-1α expression. It was reported that HIF-1α protein synthesis is regulated by the activation of MAPK signaling pathway (Blouin et al., 2004; Olson and van der Vliet, 2011). These pathways subsequently mediated eukaryotic translation initiation factor 4E (eIF-4E) and the ribosomal S6 protein to increase the expression of HIF-1α protein (Semenza, 2003). Thus, we focused further studies on MAPK signaling. First, we investigated the role of STS on the activation of MAPK signaling. Previous studies have demonstrated that MAPK signaling play important roles in regulating COPD-associated phenotypes, including chronic inflammation and cytokine expression (Bewley et al., 2016). Our present study also showed that the phosphorylation of ERK, p38 MAPK and JNK was significantly increased in CSE-primed RAW 264.7 cells, suggesting that MAPK signaling were activated in macrophages. However, the enhancement of phosphorylated ERK, p38 MAPK and JNK was significantly inhibited by STS. Further, treatment with ERK, p38 MAPK or JNK inhibitor, respectively, successfully repressed CSE-induced upregulation of TNF-α, IL-1β and HO-1 (data not shown here). Taken together, these results demonstrated that inhibition of MAPK signaling contributed to the suppression effect of STS on CS-induced inflammatory response and oxidative stress. Second, we examined whether the inactivation of MAPK signaling accounted for the suppression of STS on HIF-1α expression. Our Western blot results showed that treatment with PD98059 (ERK inhibitor), SP 600125 (JNK inhibitor) or SB 203580 (P38 MAPK inhibitor) significantly repressed HIF-1α expression, revealing that STS blocked CS-induced HIF-1α activation via the suppression of the MAPK pathways. Additionally, previous studies reported that various inflammatory mediators can interact with their cognate receptor tyrosine kinase, leading to activation of MAPK pathways (Gorlach, 2009). Several pro-inflammatory mediators, such as TNF-α and IL-1β, have been demonstrated to induce HIF-1α protein expression through this mechanism (Frede et al., 2005). Interestingly, our results showed that treatment with STS significantly inhibited CS-induced upregulation of TNF-α and IL-1β *in vivo* and *in vitro.* Thus, we speculated that STS might inhibit MAPK pathway via suppression of TNF-α/IL-1β production.

**FIGURE 9:**
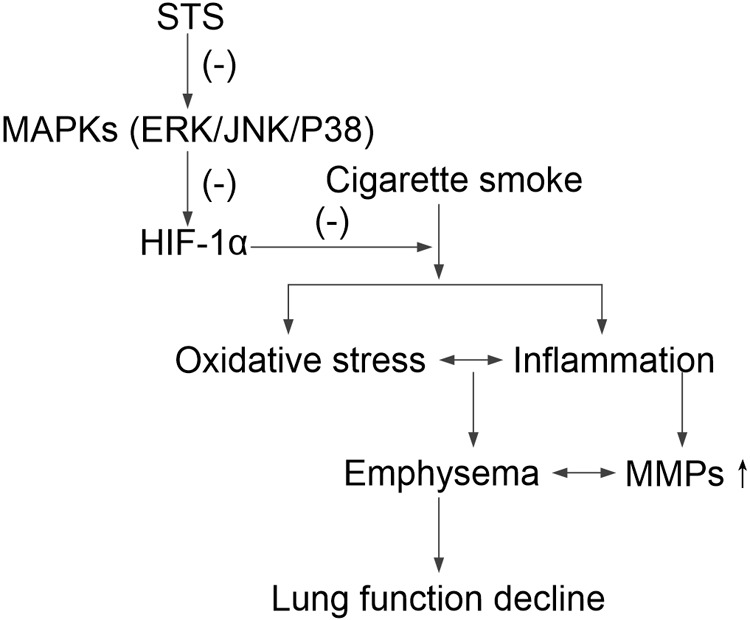
A schematic pathway illustrating the protective effects of STS against CS-induced COPD in mice. STS decreases the phosphorylation of ERK, p38 MAPK and JNK, which is responsible for HIF-1α down-regulation, resulting in protection against inflammatory responses, oxidative stress, emphysema and lung function decline in CS-exposed lungs.

Further, we investigated whether STS could affect HIF-1α protein degradation. Previous studies have demonstrated that the level of HIF-1α protein is regulated by post-translational degradation and chronic CSE exposure increased HIF-1α stabilization (Sun et al., 2011). In this study, we found that the half-life of HIF-1α protein was significantly shortened in the presence of STS, indicating that STS promoted HIF-1α protein degradation. A schematic diagram of STS targets presented in **Figure 9**.

In the present study, we confirmed that STS could provide a new option for the treatment of COPD, whose advantage exists in the following two aspects. First, the safety of STS is established by its long clinical experience of application with rarely reported adverse effects (Yang et al., 2008; Jiang et al., 2016). Second, it is economical for patients due to its high content in some plants such as *Salvia miltiorrhiza* Bunge and *S. japonica* Thunb. However, the current study has several limitations. First, this study only investigated the effects of a single dose of STS on CS-induced COPD in mice. Thus additional work is required to examine a dose response curve to evaluate the effects of different doses of STS on CS–induced oxidative stress and inflammation. Second, this study only observed the preventive effects of STS on CS-induced COPD mice. Whether STS could limit the progression of established COPD needs to be documented. Third, in addition to Tanshinone IIA, there are a number of other components such as salvianolic acid A, tanshinone I, and cryptotanshinone in Danshen extract, which have been reported to have anti-oxidative or anti-inflammatory function, and among them salvianolic acid A was considered as one of the strongest antioxidant compounds so far discovered in the world (Chen et al., 2012; Chen and Cui, 2017; Yuan et al., 2017). In the future, we will continue to look for other potential components in Danshen extract.

In summary, we demonstrated that STS alleviated the severity of CS-induced COPD in mice, attenuated inflammation and oxidative stress in macrophages, and reduced the production of TNF-α and IL-1β *in vivo* and *in vitro*, which were associated with the suppression of ERK, p38 and JNK MAPK phosphorylation and subsequently inhibition of HIF-1α signaling pathway. Given its high safety and cheapness, we propose that STS could probably be used clinically to treat COPD.

## Author Contributions

WL, RG, and DS conceived the project and designed the experiments. RG wrote the manuscript. WL, RG, JW, GX, and TW edited the manuscript. ZiL, MD, RG, DL, QY, ZhL, YC, ZC, CZ, and XL performed the experiments. LZ, LD, HZ, and RG conducted the data analysis. All authors have read and approved the final manuscript.

## Conflict of Interest Statement

The authors declare that the research was conducted in the absence of any commercial or financial relationships that could be construed as a potential conflict of interest.
